# Relationship Between Soluble ST2 Level and Chronic Thromboembolic Pulmonary Hypertension (CTEPH) in Acute Pulmonary Embolism (PE) Patients

**DOI:** 10.7759/cureus.42449

**Published:** 2023-07-25

**Authors:** Murat Kerkütlüoğlu, Hakan Gunes, Nurhan Atilla, Enes Celik, Musa Dagli, Muhammed Seyithanoglu

**Affiliations:** 1 Cardiology, Faculty of Medicine, Kahramanmaras Sutcu Imam University, Kahramanmaras, TUR; 2 Chest Disease, Faculty of Medicine, Kahramanmaras Sutcu Imam University, Kahramanmaras, TUR; 3 Biochemistry, Faculty of Medicine, Kahramanmaras Sutcu Imam University, Kahramanmaras, TUR

**Keywords:** immunology, pulmonary arterial hypertension, soluble st-2, pulmonary embolism, chronic thromboembolic pulmonary hypertension

## Abstract

Background: Chronic thromboembolic pulmonary hypertension (CTEPH) is a disease characterized by right heart failure following recurrent pulmonary embolism (PE). It is important to know the predictors of the development of CTEPH after PE as it is a treatable cause of pulmonary arterial hypertension. Soluble ST2 is a biomarker closely associated with heart failure and the inflammatory process. The aim of this study was to investigate the relationship between sST2 level and the development of CTEPH in patients with PE.

Methodology: Baseline characteristics, electrocardiographic findings, laboratory findings, transthoracic echocardiography (TTE) findings, location, and extent of involvement in CT pulmonary angiography were recorded in 100 patients with acute PE included in our prospective study. Treatment modalities and treatment durations were followed. Ventilation-perfusion scintigraphy was performed in patients with a systolic pulmonary artery pressure (sPAP) of 35 mmHg or more on TTE and residual thrombus on CT pulmonary angiography after at least three months of anticoagulant use. In the case of findings compatible with CTEPH in these examinations, patients were diagnosed with CTEPH by right heart catheterization. The sST2 levels obtained from all patients at admission were evaluated between the groups of patients with and without CTEPH.

Results: CTEPH was observed in 11 of the 100 patients who participated in the trial, with a median follow-up of 284 ± 60 days. The mean age of the 11 patients with CTEPH was 67 ± 10 years; five were males and six were females. The mean age of 89 patients without CTEPH was 65 ± 18 years, 36 were males and 53 were females. The sST2 values of the group with CTEPH were found to be statistically significantly higher than those of patients without CTEPH [193.7 (184.3-244.7) vs 58.6 (29.5-122.9) p=0.020]. This receiver operating characteristic (ROC) curve shows that the optimal cutoff point of sST2 levels in the prediction of CTEPH was > 157.4 with specificity of 83.7% and sensitivity of 81.8% (area under the curve = 0.783; 95% CI, 1.005-1.027; p < 0.001).

Conclusion: In acute PE patients, sST2 levels may be a useful biomarker to predict the development of CTEPH.

## Introduction

One of the main causes of pulmonary hypertension (PH) is chronic thromboembolic pulmonary hypertension (CTEPH), which is defined as pulmonary hypertension linked to insufficient resolution of recurrent PE [1]. A mismatch defect on ventilation-perfusion scintigraphy and a mean pulmonary artery pressure of > 25 mm Hg, despite proper anticoagulant treatment for three months after PE, are diagnostic indicators of CTEPH, despite the difficult diagnostic method [2]. Although pulmonary arterial hypertension (PAH) is mostly treated with medicines tailored to the condition, none of these treatments are curative [3]. In carefully chosen individuals, pulmonary thromboendarterectomy (PTE) for CTEPH offers a curative alternative with outstanding long-term outcomes [4]. Markers that predict the development of CTEPH after PE are important for the treatment and diagnosis of this disease.

sST2, a new biomarker for cardiac stress, fibrosis, and inflammation, belongs to the interleukin (IL-1) receptor family and has both transmembrane (ST2L) and soluble (sST2) isoforms. When secreted into circulation, ST2L's soluble form, or sST2, serves as an IL-33 trap receptor [5]. Previous studies have demonstrated that certain inflammatory and cardiac disorders are associated with elevated blood levels of sST2 [6]. Moreover, it has been discovered to be a clinically practical predictive indicator for patients with heart failure [7]. One of the most critical issues for patients with CTEPH is right heart failure. Progressive right ventricular (RV) failure may develop over time because of elevated pulmonary arterial pressure caused by increased pulmonary vascular resistance [8]. Interleukin-33, the circulating sST2 ligand, was demonstrated in a previous study to potentially contribute to vascular remodeling of the pulmonary endothelium in idiopathic pulmonary arterial hypertension (iPAH) [9]. Moreover, sST2 levels are associated with RV dysfunction in patients with PAH [10]. It is unknown whether sST2 levels and CTEPH in patients are correlated. This study aimed to examine the relationship between sST2 levels and CTEPH in patients with PE.

## Materials and methods

Study design and patient selection

In our prospective observational study, patients with PE admitted to the Kahramanmaras Sutcu Imam University Hospital were included. Individuals suspected of having PE underwent a conventional diagnostic process (2019 ESC Guidelines for the Diagnosis and Management of Acute Pulmonary Embolism, developed in collaboration with the European Respiratory Society). The inclusion criteria for the study were patients with PE confirmed by transthoracic echocardiography (TTE) and computed tomography angiography of the pulmonary arteries (CTPA). 

All patients with PE were enrolled regardless of their history of venous thromboembolism (VTE). Study exclusion criteria: being under the age of 18, presence of active malignancy or infection, active connective tissue disease that triggers inflammation, history of recurrent PE, heart failure diagnosis, history of chronic thyroid disease treatment, portopulmonary hypertension diagnosis, the data planned for the study could not be obtained in full, patient non-compliance, such as discontinuation of medical treatment during follow-up, and failure to establish communication with patients, which is necessary for the determination of secondary outcomes.

A total of 147 consecutive patients were included in this study. The study excluded 47 patients who did not show up for follow-up appointments, did not take their prescribed medications, or died within the first three months. Researchers who were blinded to the biomarker levels recorded the clinical findings, demographic data, laboratory results, treatment, and follow-up of 100 patients who were part of the trial using a standardized questionnaire. Blood samples were also drawn to measure sST2 levels. Immediately following intake, the blood samples were centrifuged and stored at -80°C for analysis. When the patient was presented to the emergency room, a similar program was used to calculate the pulmonary embolism severity index (PESI) score. TTE and electrocardiography (ECG) were performed on admission. The outcomes of TTE, lower extremity venous Doppler USG, location, and degree of involvement on CTPA, ventilation/perfusion (V/P) scintigraphy results, PESI score, therapy methods, and treatment duration were recorded.

According to the most recent PE recommendations, patients who were in the non-massive (low-risk) PE group and had no contraindications began low-molecular-weight heparin (LMWH) treatment at an appropriate dose based on weight. Within the first 24 h of treatment, warfarin was introduced (unless there was an obstacle). After taking LMWH and warfarin together for at least five days and the international normalized ratio (INR) readings remaining within the therapeutic range (between 2 and 3), the LMWH medication was stopped. The INR was also determined. LMWH was used to maintain maintenance therapy in individuals unable to take warfarin. Patients who were unable to attain an effective INR level received new oral anticoagulant (NOAC) medication.

After ruling out any contraindications and obtaining approval from the patients and their loved ones, the patients in the large PE group who had a shock, hypotension, severe hypoxemia, tachycardia, and right heart failure on TTE received thrombolytic therapy (tPA: 100 mg/2 h IV infusion). As with other patients, switching to maintenance medication was accomplished with warfarin or LMWH (NOAC if an effective INR was not established). All the patients received anticoagulant treatment for more than three months. After acute PE, TTE controls were performed three months later, and in patients who had a high or moderate likelihood of developing PAH, right cardiac catheterization and CTPA were used to explore the diagnosis of CTEPH. Written informed consent was obtained from each patient, and the study was approved by the Ethics Committee of the Faculty of Medicine, Kahramanmaras Sutcu Imam University (2019/11-184).

Biomarker assays

Peripheral vein sampling was used to collect samples in EDTA-containing tubes, which were then immediately centrifuged and stored at -80°C for later analysis. Aspect-PLUS ST2 Rapid Test, TM, a highly sensitive sandwich monoclonal immunoassay with a lower limit of detection of 12.5 ng/mL, an upper limit of detection of 250 ng/mL, an intra-assay coefficient of variation of 10.4%, and an inter-assay coefficient of variation of 13.6%, was used to measure the levels of sST2 in baseline samples.

Chronic thromboembolic pulmonary hypertension: diagnostic algorithm

The same physician, who was blinded to the research each time, evaluated the TTE using 2-D, conventional, and tissue Doppler ECHO with a GE Vivid E90 instrument (GE Healthcare, Milwaukee, WI) and a 5 MHz transducer probe. The right heart chamber size, septal deviation, pericardial effusion, RV/left ventricular (LV) diameter ratio, and other findings were evaluated and reported. Ejection fraction (EF) and systolic pulmonary artery pressure (sPAP) measurements, tricuspid annular plane systolic excursion (TAPSE), and other findings were recorded. TAPSE was calculated by observing the amplitude of motion at the intersection between the right ventricle and the tricuspid annulus. In the presence of tricuspid regurgitation, the sPAP was computed using Doppler echocardiography and the streamlined Bernoulli equation. Right heart function was assessed using measurements of the right atrium and RV size, RV fractional area change, TAPSE, RV S', pulmonary artery diameter, pulmonary acceleration time, and sPAB. Patients with a moderate-to-high probability of PAH (sPAB 36 mmHg with additional TTE results or sPAB >36 mmHg) were assessed for CTEPH according to the TTE criteria in the most recent PAH guidelines.

A multidetector (64-slice) CT scan was performed using a common contrast agent (Aquilion One; Toshiba Medical Systems, Ohtawara, Japan). Chest sections (0.5 mm) were obtained to diagnose and rule out PE. The tube voltage was 120 kV, the tube current was 300-350 mA, and the rotation duration was 0.5 s. Images were taken after an IV catheter automatically delivered 80-100 mL of contrast medium at a rate of 4 mL/s while the patient held their breath for 10-12 s. The radiologists reported the images. If at least one filling defect in the pulmonary arteries and their branches were noted, PE was considered present. If findings consistent with CTEPH (intraluminal filling defect, thrombus running parallel to the vessel wall and making a wide angle, sequelae such as web and band, mosaic perfusion, recanalization, and calcification within the thrombus) were reported in patients with suspected CTEPH, they were assumed to have the disease. Patients with CTPA results consistent with those of CTEPH were scheduled for right cardiac catheterization. After inserting a 6-French sheath into the right femoral vein, a Swan-Ganz catheter was used to measure the hemodynamics of the right atrium, right ventricle, left pulmonary artery, and right pulmonary artery. During right cardiac catheterization, the diagnosis of CTEPH has been confirmed if the mean pulmonary artery pressure was less than 25 mmHg and the pulmonary capillary tip pressure was less than 15 mmHg, and disease involvement was demonstrated by pulmonary angiography. If sPAB was >50 mmHg after at least three months of anticoagulant use and findings compatible with CTEPH were observed on CTPA, patients in whom written consent for right heart catheterization could not be obtained or in whom invasive intervention was not deemed appropriate due to hemodynamic impairment and comorbidities were diagnosed with CTEPH.

Statistical analysis

Statistical analyses were conducted using the SPSS program version 24 (SPSS Inc., Chicago, IL), and a p-value of 0.05 was deemed statistically significant for interpreting the study's findings. Continuous data are expressed as means, standard deviations, or medians, and categorical data are expressed as percentages (min-max). Categorical variables were compared between the groups using Fisher's exact test and Pearson's chi-square test. The Mann-Whitney U test and Student’s t-test were used to determine whether the average differences between the groups were statistically significant. Correlations were assessed using Spearman's correlation test. Receiver operating characteristic (ROC) curve analysis was used to establish the cutoff value of sST2 for diagnosing CTEPH. Using MedCalc (v12.7.8) (MedCalc Software bvba, Ostend, Belgium), ROC curve analysis was performed. Using univariate analysis, we determined how the different parameters were related to CTEPH. Using a forward stepwise approach, variables that were statistically significant in the univariate analysis and additional possible confounders were added to a multivariate logistic regression model to identify independent risk factors for CTEPH.

## Results

Chronic thromboembolic pulmonary hypertension was observed in 11 of the 100 patients who participated in the trial, with a median follow-up of 284 ± 60 days. The average age of the 11 patients with CTEPH was 67 ± 10 years; five of the patients were males and six were females. Of the 89 patients without CTEPH, 36 were men and 53 were women, with a mean age of 65 ± 18 years. The demographics and concomitant chronic illnesses (smoking, hypertension, diabetes, hyperlipidemia, and chronic renal failure) of the two groups were comparable (Table 1). Chest discomfort was found to be statistically significantly more frequent in the CTEPH group when both groups were evaluated in terms of symptoms at presentation [9 (81%) vs. 29 (32%), p = 0.020]. When the results of hospitalization were analyzed, the group with CTEPH had higher diastolic blood pressure (65 ± 7 vs. 58 ± 10, p = 0.020). Additional findings in both groups were comparable (Table 1).

**Table 1:** Demographic characteristics, presenting complaints, and presenting findings of PE patients. CTPEH, chronic thromboembolic pulmonary hypertension; PESI, pulmonary embolism severity index; IQR, inter quartile range

	CTEPH (n=11)	Non-CTEPH (n=89)	p Value
Demographic Characteristics			
Age (years), mean, ± SD	67 ± 10	65 ± 18	0.606
Gender (M/F)	5/6	36/53	0.318
Height (cm), mean, ±SD	167 ± 6	164 ± 7	0.262
Weight (kg), mean, ±SD	75.6 ± 11.8	76.3 ±10.6	0.690
Hypertension, n, (%)	8 (72%)	43 (48%)	0.122
Diabetes mellitus, n, (%)	3 (27%)	18 (20%)	0.691
Hyperlipidemia, n, (%)	4 (36%)	28 (31%)	0.735
Chronic renal Impairment, n, (%)	1 (9%)	11 (12%)	0.624
Smoking, n, (%)	1 (9%)	21 (23%)	0.450
Application complaints			
Chest pain, n, (%)	9 (81%)	29 (32%)	0.020
Syncope, n, (%)	4 (36%)	19 (21%)	0.259
Dyspnea, n, (%)	11 (100%)	87 (98%)	0.797
Lower extremity edema, n, (%)	7 (63%)	28 (31%)	0.410
Pain in lower extremity, n, (%)	9 (81%)	28 (31%)	0.127
Application findings			
Systolic blood pressure (mmHg), mean, ±SD	100 ± 11	102 ± 16	0.716
Diastolic blood pressure (mmHg), mean, ±SD	65 ± 7	58 ± 10	0.020
Pulse rate (beats/min), mean,±SD	120 ± 15	115 ± 17	0.355
O2 saturation (%), mean, ±SD	79 ± 7	81 ± 11	0.512
Body temperature (degree Celsius), mean,± SD	37 ± 1	37 ± 5	0.819
Respiratory rate /min, mean, ±SD	33 ± 4	30 ± 3	0.280
PESI SCORE, median, (IQR)	166 (145-221)	132 (91-213)	0.91

The ECG results in both groups were comparable when TTE parameters were evaluated. The RV diameter of patients with CTEPH was substantially larger than that of individuals without CTEPH when TTE results were evaluated (41.34 ± 5.12 vs. 36.01 ± 6.45; p=0.01). Patients with CTEPH had significantly lower values for RV fractional area changes, TAPSE, and RV S' (33.0 ± 5.79 vs. 38.15 ± 7.71, p=0.030; 12.39 ± 1.94 vs. 15.62 ± 3.73, p<0.001; 11.02 ± 1.13 vs. 12.26 ± 1.49, p=0.010; respectively) than those without CTEPH. Tricuspid regurgitation jet flow and sPAP values were considerably greater in patients with CTEPH (2.81 ± 0.33 vs. 2.52 ± 0.37, p=0.015; 46.55 ± 7.85 vs. 37.40 ± 9.90, p=0.04; respectively). Patients with CTEPH had a significantly higher RA area (20.56 ± 3.36 vs. 17.20 ± 4.79, p=0.026). The patient group with CTEPH had pulmonary artery diameters that were significantly greater than those of the group without CTEPH (17.20 ± 4.79 vs. 27.66 ± 4.58, p = 0.009). Pulmonary acceleration time was considerably less (88.55 ± 7.51 vs. 98.16 ± 12.00, p = 0.02) (Table 2).

The presence of thrombus in the right pulmonary artery was substantially more common in patients who developed CTEPH when the admission CTPA findings of the patients were assessed [8 (72%) vs. 31 (33%), p = 0.019]. The additional findings in both groups were comparable (Table 2).

**Table 2:** ECG characteristics, TTE parameters, and CTPA findings of PE patients at admission. CTPEH, chronic thromboembolic pulmonary hypertension; RBBB, right bundle branch block; TTE, transthoracic echocardiography; RV, right ventricle; TAPSE, tricuspid annular plane systolic excursion; sPAP, systolic pulmonary artery pressure; PA, pulmonary artery; CTPA, computed tomography of pulmonary artery

	CTEPH (n=11)	Non-CTEPH (n=89)	p Value
Application ECG features			
Sinus tachycardia, n, (%)	0 (0%)	29 (32.5%)	0.310
Atrial dysrhythmia, n, (%)	4 (36.3%)	21 (23.5%)	0.259
RBBB, n, (%)	7(63.6%)	25 (28%)	0.211
S1-Q3-T3, n, (%)	1 (9.09%)	15 (16.8%)	0.461
Application TTE parameters			
RV diameter (mm), mean, SD	41.34 ± 5.12	36.01 ± 6.45	0.010
RV fractional area change (%), mean, SD	33.0 ± 5.79	38.15 ± 7.71	0.030
RV S' (cm/s), mean, SD	11.02 ± 1.13	12.26 ± 1.49	0.010
TAPSE (mm), mean, SD	12.39 ± 1.94	15.62 ± 3.73	<0.001
Tricuspid regurgitation flow velocity (m/s), mean, SD	2.81 ± 0.33	2.52 ± 0.37	0.015
sPAP (mmHg), mean, SD	46.55 ± 7.85	37.40 ± 9.90	0.040
PA diameter (mm), mean, SD	31.42 ± 2.84	27.66 ± 4.58	0.009
Pulmonary flow velocity (m/s), mean, SD	1.03 ± 0.01	1.01 ± 0.02	0.014
Pulmonary acceleration time (ms), mean, SD	88.55 ± 7.51	98.16 ± 12.00	0.020
Right atrium area (cm2), mean, SD	20.56 ± 3.36	17.20 ± 4.79	0.026
CTPA findings			
Main pulmonary artery, n, (%)	1 (9%)	3 (3%)	0.368
Right PA, n, (%)	8 (72%)	31 (33%)	0.019
Left PA, n, (%)	3 (27%)	32 (35%)	0.746
Right segmentary PA, n, (%)	6 (55%)	35 (38%)	0.339
Left segmentary PA, n, (%)	6 (55%)	40 (43%)	0.534
Right subsegmentary PA, n, (%)	6 (55%)	26 (28%)	0.092
Left subsegmentary PA, n, (%)	4 (36%)	29 (32%)	0.742

According to test results, patients with CTEPH had significantly higher sST2 levels [193.7 (184.3-244.7) vs. 58.6 (29.5-122.9) p=0.020]. Other laboratory results in both groups were comparable (Table 3).

**Table 3:** Admission laboratory findings of PE patients. CTPEH, chronic thromboembolic pulmonary hypertension; BUN, blood urea nitrogen; ALT, alanine aminotransferase; AST, aspartate aminotransferase; GGT, gamma glutamil transferase; LDH, lactate dehydrogenase; WBC, white blood cell; RBC, red blood cell; RDW, red cell distribution width; MPV, mean platelet volume; CK-MB, creatine kinase-myoglobin binding; BNP, brain natriuretic peptide; CRP, C-reactive protein; PE, pulmonary embolism; IQR, inter quartile range; SD, standard deviation

	CTEPH (n=11)	Non-CTEPH (n=89)	p Value
Glucose (mg/dL)	126 (102-151)	121 (96-175)	0.161
BUN (mg/L)	32 (12-34)	18.5 (13-28)	0.476
Creatine (mg/L), median, (IQR)	0.84 (0.76-1.2)	0.82 (0.58-1.02)	0.390
Sodium (mmol/L) , mean, SD	138 ± 4	140 ± 5	0.162
Potassium (mmol/L) , mean, SD	4.4 ± 0.4	4.1 ± 0.5	0.901
Chlorine (mmol/L), mean, SD	99.2 ± 5.6	102.0 ± 6.1	0.157
Calcium (mg/dL), mean, SD	8.7 ± 0.5	8.3 ± 0.8	0.107
Magnesium (mg/dL), mean, SD	1.9 ± 0.3	1.8 ± 0.3	0.497
ALT (U/L) , median, (IQR)	18 (12-49)	21 (12-36)	0.841
AST (U/L), median, (IQR)	27 (23-38)	27 (20-36)	0.734
GGT (U/L), median, (IQR)	56 (21-69)	39 (18-74)	0.605
LDH (mg/dL) , median, (IQR)	259 (213-352)	294 (214-398)	0.487
Total protein (g/L), mean, SD	67.9 ± 8.7	61.9 ± 8.2	0.053
Albumin (g/L), mean, SD	36.9 ± 5.6	33.2 ± 6.1	0.067
Hemoglobin (g/dL), mean, SD	13.3 ± 2.1	11.4 ± 2.1	0.150
Hematocrit (%), mean, SD	41.1 ± 6.1	35.4 ± 5.9	0.130
WBC (10^9/L), mean, SD	10.5 ± 4.3	10.5 ± 5.5	0.979
RBC (10^6 U/L), mean, SD	5.1 ± 0.6	4.1 ± 0.71	0.001
Platelet (10^9/L), median, (IQR)	252 (210-325)	266 (188-351)	0.472
RDW (%), mean, SD	49.3 ± 8.1	48.5 ± 9.3	0.795
MPV (fl), mean, SD	11.0 ± 0.7	10.1 ±1.3	0.060
pro-BNP (ng/L), median, (IQR)	2450 (1020-3080)	590 (267-1650)	0.129
D-Dimer (mg/L), median, (IQR)	3.6 (1.06-5.54)	4.7 (2.2-12.8)	0.252
CK-MB (ng/dL), median, (IQR)	5.1 (2-6.1)	2 (0.96-5.1)	0.075
Troponin (ng/dL), median, (IQR)	0.20 (0.02-1.98)	0.04 (0.01-0.6)	0.165
CRP (mg/L), median, (IQR)	17 (7-27)	63 (21-108)	0.330
Prokalsitonin (mg/L), mean, SD	0.2 ± 0.1	0.7 ± 1.8	0.376
Soluble ST2 (ng/mL), median, (IQR)	193.7 (184.3-244.7)	58.6 (29.5-122.9)	0.020

The RV diameter, pulmonary artery pressure, pulmonary artery diameter, and right atrial area were all positively correlated with serum sST2, whereas the RV fractional area change, RV S', TAPSE, and pulmonary artery axialization time were negatively correlated (Table 4).

**Table 4:** sST2 correlation coefficients. CK-MB, creatine kinase-myoglobin binding; RDW, red cell distribution width; BUN, blood urea nitrogen; BNP, brain natriuretic peptide; RV, right ventricle; sPAP, systolic pulmonary artery pressure; PA, pulmonary artery; FAC, fractional area change; TAPSE, tricuspid annular plane systolic excursion

	r	p
Troponin	0.218	0.027
CK-MB	0.298	0.002
RDW	0.312	0.001
Age	0.241	0.014
Glucose	0.270	0.006
BUN	0.373	<0.001
Creatinine	0.274	0.005
Prokalsitonin	0.416	<0.001
pro-BNP	0.438	<0.001
RV diameter	0.365	<0.001
sPAP	0.405	<0.001
PA diameter	0.311	0.001
RA area	0.371	<0.001
PA acceleration time	-0.382	<0.001
RV FAC	-0.413	<0.001
RV S’	-0.351	<0.001
TAPSE	-0.423	<0.001

**Figure 1:**
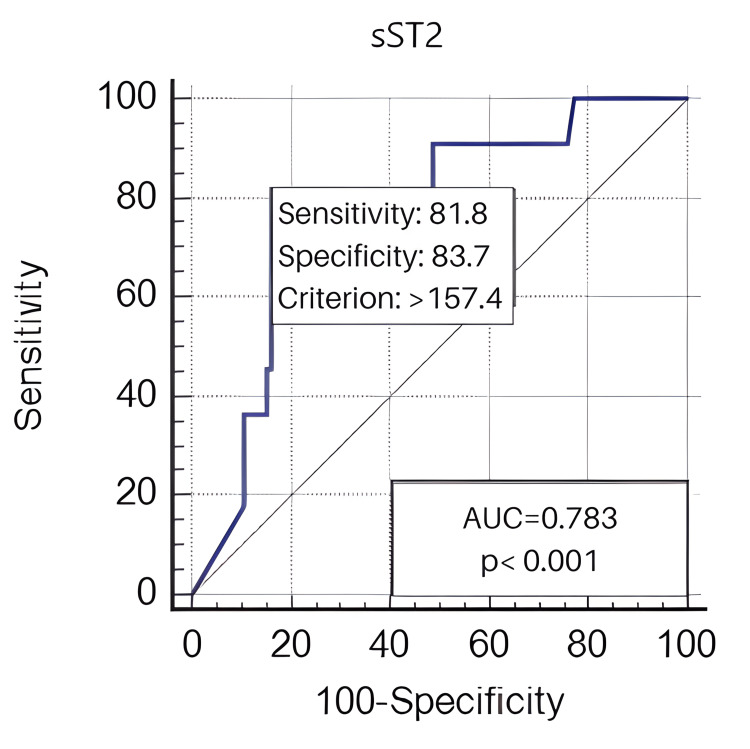
ROC curve of sST2 predicts CTEPH. ROC, receiver operator characteristic; CTEPH, chronic thromboembolic pulmonary hypertension; sST2, soluble sT2

The optimal cutoff value for sST2 levels in the prediction of CTEPH was > 157.4 with a specificity of 83.7% and sensitivity of 81.8% (area under the curve = 0.783; 95% CI, 1.005-1.027; p < 0.001), according to the ROC curve (Figure 1).

In the multiple logistic regression model using a backward stepwise method, sST2 levels (OR = 1.016, 95% CI: 1.005-1.027; p = 0.003), sPAP (OR = 1.107, 95% CI: 1.005-1.219; p = 0.039), and chest pain (OR = 0.056, 95% CI: 0.008-0.399; p = 0.004) still remained significant predictors of CTEPH after adjusting for the confounding variables, which were either found to be statistically significant in the univariate analysis or for the variables correlated with ST2 levels (Tables 5-6).

**Table 5:** Results of univariate analysis of predictive factors for the development of CTEPH in PE patients. sPAP, systolic pulmonary artery pressure; PA, pulmonary artery; RV, right ventricle; RA, right atrium; CK-MB, creatine kinase myoglobin binding; RDW, red cell distribution width; BUN, blood urea nitrogen; BNP, brain natriuretic peptide; CTEPH, chronic thromboembolic pulmonary hypertension; TAPSE, tricuspid annular plane systolic excursion

Variables	B	SE	Wald	p	OR	CI
sST-2	0.014	0.004	10.522	0.001	1.014	1.005-1.022
Chest pain	2.28	0.813	7.858	0.005	0.102	0.021-0.504
sPAP	0.080	0.030	7.029	0.008	1.084	1.021-1.150
Right PA involvement	1.658	0.712	5.420	0.02	5.24	1.300-21.184
RV diameter	0.119	0.049	5.899	0.015	1.126	1.023-1.240
RV FAD	0.078	0.039	4.119	0.042	0.925	0.857-0.997
RV S'	0.556	0.230	5.826	0.016	0.574	0.365-0.901
TAPSE	0.256	0.101	6.422	0.011	0.774	0.635-0.944
Incoming diastolic blood pressure	0.06	0.031	3.636	0.057	1.061	0.998-1.129
PA diameter	0.183	0.075	5.994	0.014	1.201	1.037-1.390
PA axis. Time	0.069	0.029	5.678	0.017	0.933	0.882-0.988
RA area	0.140	0.066	4.505	0.034	1.150	1.011-1.309
Variables correlated with sST2						
Variables	B	S.E.	Wald	p	OR	CI
Troponin	0.444	0.272	2.66	0.103	1.559	0.914-2.658
CK-MB	0.136	0.093	2.130	0.144	1.146	0.954-1.377
RDW	0.009	0.033	0.069	0.792	1.009	0.945-1.077
Age	0.007	0.019	0.123	0.726	1.007	0.970-1.045
Glucose	0.005	0.006	0.664	0.415	0.995	0.984-1.007
BUN	0.010	0.017	0.331	0.565	1.010	0.976-1.045
Creatinine	0.447	0.412	1.181	0.277	1.564	0.698-3.506
Prokalsitonin	0.435	0.575	0.571	0.450	0.647	0.210-1.999
Pro-BNP	0.00	0.00	0.171	0.680	1.00	1.00-1.00

**Table 6:** Results of multivariate analysis of predictive factors for the development of CTEPH in PE patients. CTEPH, chronic thromboembolic pulmonary hypertension; PE, pulmonary embolism; sPAP, systolic pulmonary artery pressure; SE, standard error; OR, odds ratio; CI, confidence interval

Variables	B	SE	Wald	p	OR	CI
sST-2	0.016	0.006	8.593	0.003	1.016	1.005-1.027
Chest pain	2.885	1.004	8.261	0.004	0.056	0.008-0.399
sPAP	0.102	0.049	4.265	0.039	1.107	1.005-1.219

## Discussion

To our knowledge, this prospective follow-up study is the first to investigate the relationship between sST2 levels and CTEPH in acute PE patients. In this study, sST2 levels were associated with CTEPH in PE patients and were independent predictors of CTEPH development. In addition to serum ST2 levels, elevated sPAP and chest pain at presentation are independent predictors of CTEPH development.

The prompt identification of CTEPH has emerged as a significant obstacle in clinical practice. The European CTEPH registry has shown a significant mean diagnostic delay of 14 months, indicating a clear demonstration of this phenomenon [11]. One possible reason for this delay could be attributed to the fact that the indications of CTEPH are predominantly nonspecific. Despite the presence of relevant pulmonary hypertension, patients may remain asymptomatic or fail to report their symptoms for several months [11]. To date, there is a lack of validated, cost-effective screening tools for CTEPH. The precise prevalence of CTEPH following symptomatic acute PE remains uncertain and has been documented to vary between 0.1% and 11.8%, according to several studies [12-15]. This study revealed that the frequency of CTEPH among individuals with acute PE was 11%. The elevated prevalence of CTEPH observed in our investigation could potentially be attributed to a deficient level of patient awareness regarding anticoagulant therapy within our nation [16]. Acquiring a more accurate understanding of the frequency of CTEPH following an episode of acute PE is crucial in determining the suitable course of action for the extended care of acute PE.

sST2 is a member of the IL-1 receptor family and encodes soluble (sST2) and transmembrane (ST2L) types. sST2, the soluble form of ST-2L, is secreted into the circulation and functions as a trap receptor for IL-33 [5]. IL-33 is secreted by cells undergoing cell damage and necrosis, cells subjected to mechanical stress, and cells in the inflammatory process. IL-33 binds to ST2L, leading to inflammatory gene transcription and responses through the release of inflammatory chemokines and cytokines. The inflammatory response results in apoptosis and fibrosis. sST 2 suppresses this inflammatory response by binding to IL-33, thus creating a defense mechanism to suppress apoptosis and fibrosis [17-19]. Although the main source of sST2 circulating in healthy individuals and patients with different diseases is currently unclear, it is thought to be synthesized in many cells and tissues, including cardiomyocytes, endothelial cells, and vascular smooth muscle cells [20]. This is particularly true for cardiac disease. Experimental data suggest that mechanical stress can induce both IL-33 and sST2 expression in cardiomyocytes and fibroblasts [21]. In acute and chronic heart failure, sST2 levels have been shown to be associated with mortality and prognosis due to mechanical stress and inflammatory processes in the left ventricle [22].

Similarly, it has been demonstrated that sST2 levels are closely related to hemodynamic parameters, especially RV dysfunction, depending on the existing inflammation process and are a mortality marker in PAH patients [23].

Occlusive thrombotic material persists and advances owing to CTEPH. The persistence and spread of thrombus materials have been the subject of numerous studies. Inflammatory cells, such as CD45+ and collagen-secreting cells, are prevalent in the vascular walls of patients with CTEPH. Additionally, circulating inflammatory substances, such as tumor necrosis factor, monocyte chemoattractant protein (MCP)-1, and C-reactive protein, are elevated in CTEPH, and MCP-1 is positively associated with pulmonary vascular resistance [24]. Primary endothelial cells from human pulmonary arteries have shown evidence of endothelial dysfunction upon exposure to CXCL10, also known as interferon-induced protein-10 (IP-10) [25].

In line with this information, the formation of dead spaces in the lungs due to obstruction caused by thrombus in patients with PE causes the onset of the inflammatory process. An excessive response to this inflammatory process can lead to the development of CTEPH. Increased sST2 levels, which are closely related to the inflammatory response and suppression of the inflammatory process, may be a defense mechanism to prevent the development of CTEPH. Increased sST2 levels may be an indicator of an excessive body response to inflammation. In light of this information, the sST2 molecule may be a useful marker for predicting the development of CTEPH in PE patients. Additionally, sST2 released from the vascular endothelium during the obstructive remodeling of the pulmonary arteries in CTEPH may be released at high levels to prevent remodeling. In our study, sST2 levels were significantly higher in patients who developed CTEPH after PE and were independent predictors of CTEPH development. Another predictor identified in our study was the sPAP value. Elevated sPAP levels and RV dysfunction in patients with PE are risk factors for CTEPH development. Many studies have demonstrated that sPAP values > 50 mmHg at presentation in patients with PE are associated with persistent pulmonary hypertension [26]. Yang et al. found that a basal sPAP value of > 50 mmHg after PE was significant in terms of CTEPH development [27]. In a previous study conducted in Turkey, it was shown that in 57% of PE patients, sPAB values > 35 mmHg and RV dysfunction were higher in patients who developed CTEPH [28]. In our study, the admission sPAP value of patients who developed CTEPH was 46.55 ± 7.85 mmHg, which was significantly higher than that of patients who did not develop CTEPH. Additionally, increased sPAP increases myocardial tension, enhances the inflammatory response, and increases sST2 levels.

Another finding of our study was that chest pain at presentation predicted CTEPH. Pleuritic chest pain is a common finding in patients with PE. Inflammation in the dead space of the pulmonary bed causes chest pain. In addition, an increase in mechanical obstruction and RV pressure, an increase in RV oxygen consumption, and a decrease in the amount of oxygen supplied cause right and LV ischemia, leading to chest pain [29]. Chest pain, which is an indirect indicator of an increase in both mechanical and neurohumoral activity, may be an indicator of an excessive inflammatory response. This finding could be used as a predictor of CTEPH in patients with PE.

Our research has some limitations. The key one is that the patients who were included in the research were not examined for inflammatory cytokines, particularly IL-33. Additionally, not all of the patients in our research had their antithrombin, protein S, protein C, factor II, or factor V Leiden variants examined. The fact that sST2 levels were tested upon admission but not during the third month's follow-up constitutes another flaw. It is plausible that certain instances of CTEPH identified during subsequent evaluations may have existed at the initial assessment. Ongoing, intensive research is being conducted in this field, and recent publications have reported various radiologic findings [30]. These findings, when detected through computed tomography pulmonary angiography used for diagnosing acute PE, may increase the likelihood of pre-existing CTEPH and potentially alter the follow-up approach. Moreover, further investigations involving a greater cohort of individuals are required to establish conclusive outcomes regarding the prognostic value of sST2 in relation to the onset of CTEPH.

## Conclusions

Already known that early diagnosis of CTEPH is important, it can lead to significant morbidity and mortality if left untreated. Accordingly, monitoring sST2 levels and performing relevant diagnostic tests at regular intervals can aid in the early detection and management of CTEPH in patients with a history of PE.
